# Data-driven ergonomic risk assessment of complex hand-intensive manufacturing processes

**DOI:** 10.1038/s44172-025-00382-w

**Published:** 2025-03-12

**Authors:** Anand Krishnan, Xingjian Yang, Utsav Seth, Jonathan M. Jeyachandran, Jonathan Y. Ahn, Richard Gardner, Samuel F. Pedigo, Adriana W. Blom-Schieber, Ashis G. Banerjee, Krithika Manohar

**Affiliations:** 1 Department of Mechanical Engineering, University of Washington, Seattle, WA USA; 2 The Boeing Company, Everett, WA USA; 3 Department of Industrial & Systems Engineering, University of Washington, Seattle, WA USA

**Keywords:** Mechanical engineering, Computer science

## Abstract

Hand-intensive manufacturing processes, such as composite layup and textile draping, require significant human dexterity to accommodate task complexity. These strenuous hand motions often lead to musculoskeletal disorders and rehabilitation surgeries. Here we develop a data-driven ergonomic risk assessment system focused on hand and finger activity to better identify and address these risks in manufacturing. This system integrates a multi-modal sensor testbed that captures operator upper body pose, hand pose, and applied force data during hand-intensive composite layup tasks. We introduce the Biometric Assessment of Complete Hand (BACH) ergonomic score, which measures hand and finger risks with greater granularity than existing risk scores for upper body posture (Rapid Upper Limb Assessment, or RULA) and hand activity level (HAL). Additionally, we train machine learning models that effectively predict RULA and HAL metrics for new participants, using data collected at the University of Washington in 2023. Our assessment system, therefore, provides ergonomic interpretability of manufacturing processes, enabling targeted workplace optimizations and posture corrections to improve safety.

## Abstract (web summary)

A data-driven ergonomic risk assessment system for hand and finger activity is designed to identify issues in hand-intensive manufacturing processes. The system captures injurious activity and leverages machine learning to automate and enhance risk-scoring methods.

## Introduction

Hand-intensive manufacturing processes are essential in specialized industries such as textile draping, precision leatherwork, fine carpentry, and decorative artwork. This is particularly true in aerospace composites manufacturing, where increasing demand has surpassed the capacity of automated solutions. Although machines such as Automated Fiber Placement (AFP) machines are capable of laying large composite parts such as wing panels, they are unsuitable for creating smaller and more complex geometric components that involve intricate features like narrow channels or transitions from convex to concave surfaces, such as hat stringers^1^. These intricate parts, for now, can only be produced by skilled operators during hand layup, where workers use visual and tactile feedback to carefully position the material, apply force, and make continuous adjustments to ensure proper adhesion. This process is repeated until the required gauge thickness is achieved. Unlike automated machines, humans bring adaptability, making on-the-fly adjustments to force and motion that are crucial for such detailed tasks.

These intricate hand-intensive tasks pose significant ergonomic risks to workers, particularly due to the repetitive actions and forceful exertions required. The physical demands of hand layup, combined with spatial constraints and the need for precise adjustments, can lead to musculoskeletal disorders and long-term health issues for operators. Spatial constraints and task complexity further exacerbate these risks, as characterized by the Index of Difficulty (ID), which along with movement speed and accuracy can be used to model the challenges faced by operators^2^. In addition, age and physical capacity can impact task performance, especially for older operators who may experience reduced movement adaptability^3^. Stochastic models can further capture the complexity of these tasks by considering both environmental factors (e.g., spatial constraints, tool dimensions, etc.) and operator-specific traits (e.g., strength, reach, etc.) to assess how such variables influence task speed and difficulty^4^.

Currently, research in hand-intensive manufacturing focuses on the quality of the manufactured parts rather than assessing or mitigating the impact on the operators performing these tasks^5^. Advances in Machine Learning (ML) have been used to optimize process parameters and predict output quality in various manufacturing processes, such as textile draping and spun-lace production^6–9^. Yet, very few studies have explored the complex, adaptive motions involved in these manual processes from an ergonomic perspective^10,11^. There is a clear need for a comprehensive data-driven approach that not only captures the unique skills of human operators but also effectively assesses and predicts ergonomic risks to enhance safety and efficiency in the manufacturing workplace.

There have been notable improvements to ergonomic assessment in various contexts using ML. A comprehensive review of ML in manufacturing ergonomics is provided in ref. ^12^, which discusses models tailored to individual operators, analysis of operator-workplace interactions, and system-level ergonomic design. Other significant work includes using wearable mobile sensors to analyze the postures of construction workers, demonstrating the efficacy of non-invasive monitoring in identifying ergonomic risks^13^. Subsequent research^14^ improved this approach with ML, allowing for automated risk monitoring and enhancing the accuracy and scalability of ergonomic assessment. Wearable technologies have also been instrumental in workplace safety. For instance, wearable stretch sensors have been employed to monitor human movement and detect falls, addressing critical safety issues in workplace ergonomics. Despite these advances, challenges, such as sensor calibration and false positives, remain^15^. Additionally, surface electromyogram (sEMG) sensors have been used with ML algorithms to automatically detect injurious movements during material handling^16^. A systematic review on wearable devices in ergonomic applications^17^ highlights the opportunities and current limitations, particularly the issues of standardization and data quality.

Rapid Upper Limb Assessment (RULA)^18^ and Hand Activity Level (HAL)^19^ are widely used ergonomic evaluation techniques designed to assess musculoskeletal disorder risks, specifically focusing on the upper body and hand-intensive tasks, respectively. RULA is often adapted for automation through sensor technologies, using systems such as Kinect to estimate operator poses and conduct non-intrusive ergonomic assessments^20–23^. More recent advancements have included the use of the Kinect v2 sensor for real-time ergonomic assessments and automated RULA scoring, providing a cost-effective method to assess work postures^24^.

However, these methods face limitations, such as dependence on expert ratings, which are labor-intensive and susceptible to inter-rater variability^25^. HAL, which quantifies ergonomic risk based on task frequency and cycle time, relies on subjective evaluations, further complicating consistent and standardized assessments. The inherent limitations in using Kinect and other sensing systems, such as occlusions, accuracy issues in dynamic environments, and the challenges of tracking complex movements, reduce the precision and robustness of ergonomic evaluations. Recent approaches include using spatiotemporal convolutional networks for segmenting object manipulation actions to predict ergonomic risks^26^ and developing multi-task learning frameworks that simultaneously analyze human activity and ergonomic risk^27^. Nevertheless, achieving greater consistency and accuracy in ergonomic assessment methods remains a significant challenge, necessitating further development of data-driven solutions to address these gaps.

**Fig. 1:**
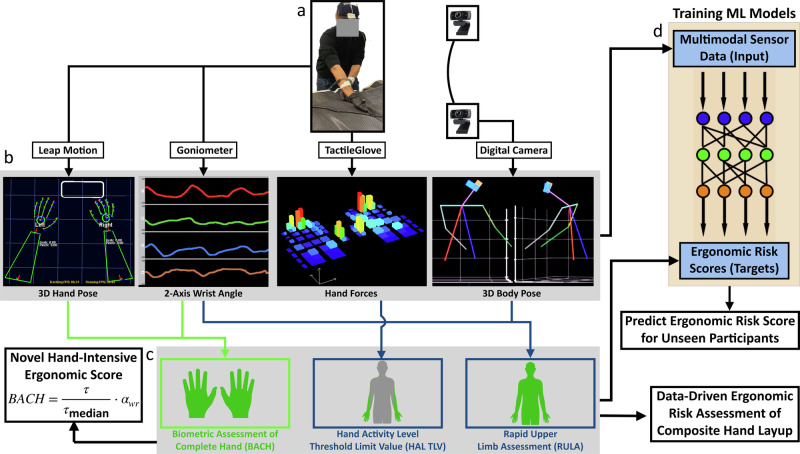
Data-driven ergonomic risk assessment pipeline. Data are collected from operators (**a**) performing hand-intensive manufacturing. Sensors (**b**) capture 3D hand and body pose, 2-axis wrist angles (flexion/extension and radial/ulnar deviation), and hand forces (bar height indicates pressure value). This multimodal sensor data enables quantitative ergonomic risk assessment (**c**) and serves as input for machine learning models (**d**) to predict risk.

This work introduces a holistic, data-driven ergonomic assessment framework for hand-intensive manufacturing, particularly focusing on composite layup tasks. Our approach involves multi-modal data collection—including 3D upper body and hand poses, as well as hand force data—combined with ergonomic scoring using industry standards such as RULA and HAL. Additionally, we introduce a novel ergonomic metric, the Biometric Assessment of Complete Hand (BACH), designed specifically to assess hand and finger-level risks. By training machine learning models to predict ergonomic scores based on multi-modal data, we aim to provide a scalable and effective solution for real-time ergonomic assessment, enhancing worker safety and reducing injury risks. The flow of multi-modal data from the sensors to the data processing pipeline for ergonomic score assessment and prediction is shown in Fig. 1. The specific contributions of this work include:

- An integrated, multi-modal sensor testbed is developed to capture data on operator pose and forces during hand layup process.
- A specialized model is presented that integrates finger and hand movements with upper body pose and hand force data to provide a comprehensive ergonomic assessment. This assessment includes industry-standard RULA and HAL scores, along with a novel BACH score.
- Automated scoring of the existing RULA and HAL risk metrics generalize well to unseen participants using machine learning.
- Empirical results show that BACH score captures hand and wrist injury risks at a higher fidelity as compared to the widely-used HAL and RULA scores.

## Methods

### Data collection

The selected sensors completely characterize the complex force motion combinations arising in hand-intensive manufacturing processes. Two digital cameras, manufactured by Nexigo (Beaverton, OR, USA), are placed at the front-left and front-right of the operator. These capture the operators’ upper body from two distinct perspectives, which is then used to reconstruct the 3D body pose.

The output from these two cameras is processed using the AlphaPose^28^ algorithm. AlphaPose is an advanced deep learning tool designed for human pose estimation, specializing in detecting and mapping human body joints in images and videos. Then, we perform bundle adjustment using a checkerboard pattern to calibrate and concurrently refine the 2D and 3D coordinates of the checkerboard corners together with the corresponding rotation and translation between the two cameras. By combining the 2D joint coordinates from AlphaPose with the rotation and translation of the two webcams, the 3D coordinates of the joints are triangulated, thereby, creating a comprehensive 3D skeletal model of the operator. The precision of the 3D reconstruction model has been evaluated, see Supplementary Section “Pose estimation accuracy verification” and Supplementary Fig. 1 for details. The remaining sensors capture arm, hand and finger motions and forces.

First, the Ultraleap Stereo IR 170 manufactured by Ultraleap (Mountain View, CA, USA) is used to record 3D hand and arm pose. This sensor records high-fidelity information about the hand and forearm joints using both infrared and visual spectrum cameras and reports 21 three-dimensional coordinates of the skeletal hand pose per hand. Next, wrist motion is captured in two axes using wired electronic goniometers, manufactured by Biometrics Ltd. (Cwmfelinfach, UK). Wrist angle is already reported by the Ultraleap IR 170 by depth sensing of the forearm and hand using infrared cameras. However, consultations with ergonomists and hand layup operators revealed that the wrist experiences the highest loads, and is likely to be the most frequently injured part. Therefore, we use this high-fidelity goniometer to ensure that wrist data are captured accurately and inter-sensor reliability is corroborated. Finger and palm forces are captured using the TactileGlove, manufactured by Pressure Profile Systems (Glasgow, UK), a pair of force-sensing gloves with 65 force-sensing elements per hand. These sensors accurately measure the location and magnitude of the forces applied by the palms and fingers.

**Fig. 2:**
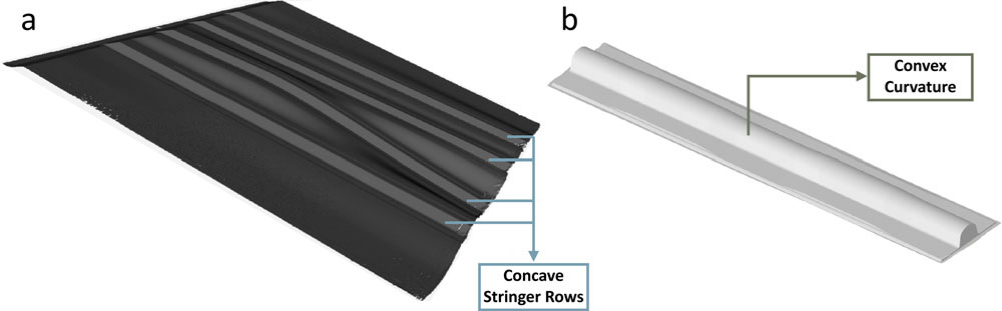
Digital scans of the two tools used for data collection. 3D scans of the Stringer tool (**a**) and Convex Mold tool (**b**) show the difference in geometry between the many concave rows of the Stringer and the convex curvature with varying radii along the length of the Convex Mold Tool.

The layup tools, materials, and shop aides cover the different types of hand motions typically performed by an operator on the factory floor. The two tools used for data collection are shown in Fig. 2. The stringer tool (Fig. 2a) is chosen for its multiple concave curvatures along its length. The operator has to use their fingers or shop aides to ensure that the carbon fiber material accurately conforms to the concave surface by applying concentric pressure to the radii to avoid bridging. In contrast, the convex mold tool (Fig. 2b) is characterized by a large convex curvature. The radius of this curvature varies along the length of the tool, requiring operators to perform smoothening motions that are characteristically almost opposite of those in the stringer tool. We also use two types of materials: a 0/90° plain-weave fabric and unidirectional tape. The material stiffness and forming behavior depend on the direction in which the ply is placed on the tool, each requiring a slightly different layup strategy. Therefore, the fabric is placed at 0° and 45° with respect to the tool, and the unidirectional material is placed at 0° and 90° with respect to the tool.

A total of 15 participants were recruited for data collection. This study complied with all relevant ethical regulations and was approved by the University of Washington IRB Committee B under ID STUDY00013896, and approved for public release (RROI #24-180410-BCA). Informed consent was obtained from all the participants prior to their participation in the study, and participant height, weight, gender, and skill level were self-reported. The participants performed layup for 15 min per tool on average. A total of 314 variables were captured at different sensing rates during each trial, which lasted for (5–15) min following a standard test procedure. The total data collection time was between (30–35) min per participant, leading to an overall 7 h of recorded data. There was significant variation in the height (mean = 169.33 cm, std. dev. = 8.23 cm) and weight (mean = 155.13 lbs., std. dev. = 28.25 lbs) of the participants. The participants’ experience level (mean = 4.08 years, std. dev. = 5.54) tended to be either low (≤3 years) or high (≥10 years). There were 7 female and 8 male participants in this study. Participants were allowed to use two shop aides in selected trials, with the expectation that they would help reduce the stress on the operators’ hands and fingers by providing a wide base and a narrow tip to access the grooves.

**Fig. 3:**
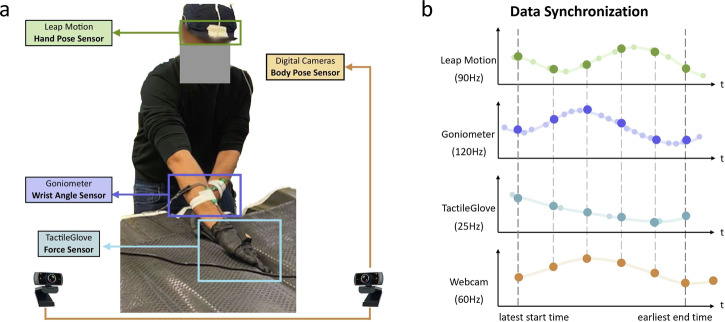
Data collection testbed and illustration of data synchronization. The sensor setup (**a**) collects data as participants perform composite hand layup, with the Leap motion sensor attached on the helmet, the goniometers attached to both wrists, the TactileGlove worn on both the hands, and two webcams (out of image) capturing stereo images. Sensors with varying frame rates are aligned and synchronized to the frame timing of the webcam (**b**). The smaller dots represent intrinsic sensing rates and the larger dots represent the interpolated and webcam-aligned data points.

The large volume and heterogeneity of the collected data, varying measurement rates of the sensors, and occasional sensor failures lead to challenges in sensor synchronization and pre-processing. Prior to initiating the trial, the two portable computers’ internal clocks are synchronized with the International Standard Time. Next, a Python script on both the computers is tasked with capturing the start and end times of data collection trials for each sensor separately. A synchronization pipeline then determines the common operational duration across all the sensors and trims their data to this unified time frame. Subsequently, the pipeline interpolates or down-samples sensor data to conform to the digital camera’s operational frame rate of 60 frames per second. The complete testbed with all the sensors for data collection is shown to the left in Fig. 3a. A schematic of this data synchronization pipeline is shown in Fig. 3b.

### Existing ergonomic assessments for upper body and hand activity

Ergonomic risk assessment of composite hand layup begins with annotation of the collected data from the operators with existing industry-standard ergonomic scores, namely RULA scores for the upper body and HAL scores for the hands. RULA is a point-based observational analysis of the joint angles and forces sustained by the upper body when executing the motion under evaluation^18^. It has the lowest score when the upper body is in a neutral posture, with penalties for deviations from this posture. The scores increase corresponding to the applied loads and decrease if there is additional support to the legs or arms. The scores for the different parts of the upper body are combined using lookup tables to provide a single score ranging from 1–7.

**Fig. 4:**
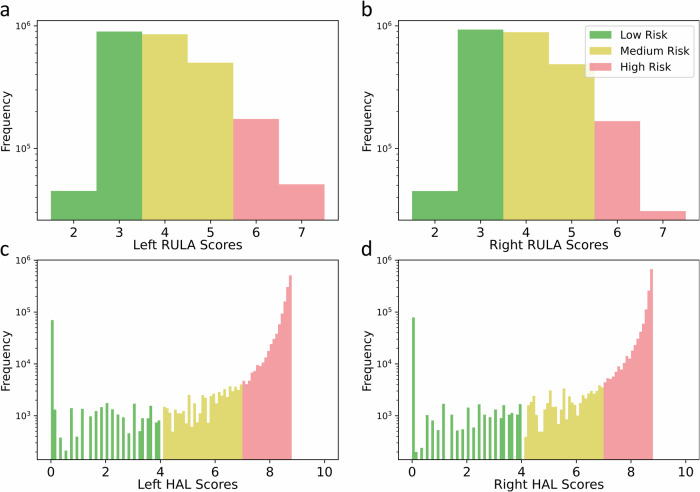
Rapid Upper Limb Assessment (RULA) and hand activity level (HAL) score distributions. Histograms of RULA (**a**, **b**) and HAL (**c**, **d**) risk scores, categorized as low (green), medium (yellow) and high (red), are presented across all participants, with the number of occurrences (frequency) on the *y*-axis. A significant portion of RULA scores fall in the medium risk range, indicating the need for operators to bend while applying force during layup. However, HAL scores most frequently fall in the high-risk category, highlighting frequent crossing of safe thresholds during composite hand layup.

RULA is calculated for each static posture in the dynamic movements comprising hand layup for each frame of data as follows. First, the 3D coordinates of the various body joints (obtained from AlphaPose) are used to compute the required arm, neck, and trunk positions and angles. Second, the wrist angles (obtained from goniometers) are used to compute the wrist position and twist. Third, the Muscle Use Score is set to 1 when the action repeats more than 4 times a minute, the Force/Load Score is set to 2 for repeated loads between 4.4 and 22 lbs., and the Leg Score is set to 1 as the legs are supported in hand layup. This information is used in the corresponding lookup Fig. 4a–c, to calculate the RULA score for a single frame. The entire dataset is annotated frame-by-frame in this manner, thereby generating a time series of RULA scores for every 3D body pose and wrist angle.

The ACGIH developed ergonomic metrics^19^ for assessing the risks of work-related distal upper extremity musculoskeletal disorders. The developed metric combines Hand Activity Level (HAL) and Normalized Peak Force (NPF) to estimate the Threshold Limit Value (TLV). HAL is a 10-point score calculated subjectively by experts viewing the performed task. These experts take into account the exertion frequency, rests, and speed of motion according to the specified guidelines. Subsequent efforts^29^ developed linear regression models and lookup tables to predict the expert-rated HAL and NPF scores. We use the estimator defined by the nonlinear regression model of Radwin et al.^30^:

1 $${{{{\rm{HAL}}}}}=6.56\,\ln \left(\frac{D{F}^{1.31}}{1+3.18{F}^{1.31}}\right),{{{{\rm{where}}}}}\,F=\frac{{{{{\rm{Exertions}}}}}}{{{{{\rm{Work}}}}}\,{{{{\rm{Time}}}}}}$$

The duty cycle *D* is defined as the ratio of work time to the total time, including rest, for a given task. Exertions are typically characterized by the speed of motion and pauses. In composite hand layup, some activities, such as repositioning material on the tool, are less strenuous than applying pressure to the surface. Therefore, exertions are calculated as the time spent above specific force thresholds, detailed below. Since data is collected only during material layup and not during rest periods, we use an average duty cycle of *D* = 75%, based on operators’ responses to a questionnaire about their working and resting times. The working period for exertion calculations is set to the mean layup motion duration, defined as Work Time = 10 s, based on observations of typical layup activity. Force thresholds are determined using the nominal forces sustained by the flexor digitorum superficialis (FDS) tendon, which facilitates flexion in the metacarpophalangeal and proximal interphalangeal joints of all fingers except the thumb. Typical force values for various hand functions are provided in Table 5 of ref. ^31^. During power grasp activities—comparable, though not identical, to hand layup—the FDS tendon typically sustains forces in the range of 4 N to 20 N. Based on discussions with ergonomists and operators’ perceived levels of effort during data collection, two force thresholds are considered: a finger force threshold of 15 N (3.3 lbs) and an overall hand force threshold of 44.8 N (10 lbs). An exertion is recorded when the load on an individual finger exceeds the finger threshold or when the total load on the hand exceeds the overall threshold:

2 $${{{{\rm{Exertions}}}}}:= \left\{\begin{array}{ll}{{{{\rm{Exertions}}}}}+1\quad &{f}_{i} > {{{{\rm{Finger}}}}}\,{{{{\rm{Force}}}}}\,{{{{\rm{Threshold}}}}}\hfill\\ {{{{\rm{Exertions}}}}}+1\quad &{\sum}_{i=1}^{5}{f}_{i} > {{{{\rm{Overall}}}}}\,{{{{\rm{Force}}}}}\,{{{{\rm{Threshold}}}}}\end{array}\right..$$

where,

$${f}_{i}=\,{\mbox{Sum of forces applied by finger i}}\,$$

The HAL score is, therefore, computed as follows. First, the forces applied over each element in a finger is summed up, and this is repeated for all the five fingers. Next, for the first ten seconds, the number of exertions is counted using Eq. (2). *F* is computed using Exertions, and subsequently the HAL score using *D* and Eq. (1). This step is repeated after sliding over the time window by a single data point and annotating the next time window with a corresponding HAL score until the last data point is reached. Therefore, the resultant HAL score is a continuous time-windowed score that is offset from the collected data by 10 s. The score is then padded with zeros at the start to ensure it is of the same length as the collected data and consistent with the other ergonomic risk scores.

Figure 4 presents histograms of RULA (4a, b) and HAL (4c, d) scores for all the participants, categorized as low risk (green), medium risk (yellow), and high risk (red). RULA scores are classified as low (0–3), medium (4,5), and high (6,7), while HAL scores are categorized as (0 < *HAL* < 4), medium (4 < *HAL* < 7), and high (*HAL* > 7).

### New score for assessing hand motion risk: BACH

The existing ergonomic risk scoring techniques have a few caveats in their assessment. RULA, for example, is not dynamic, although some adjustments exist for the total forces applied and muscle use. However, the information about the variation and location of these forces is not taken into account. RULA also focuses on the entire upper body, with no special emphasis on the hands apart from the deviations from the natural wrist position. Therefore, it cannot be used as the only ergonomic assessment of a hand-intensive process, such as hand layup. Even hand-intensive metrics such as HAL TLV need augmentation. The correlation between TLV and injury risk was studied for 908 operators in the cross-sectional assessment in ref. ^32^, which reported the prevalence of MSDs even at acceptable levels of TLV, suggesting the need for metrics that can better characterize the ergonomic risks in the hands. Informed by research indicating that wrist pressure plays a significant role in hand fatigue, chronic tendon problems, and potential injuries^33,34^, we develop the Biometric Assessment of Complete Hand (BACH) score, which focuses on the effects of hand layup motions in the wrist area.

The BACH score is derived by integrating data from three sensors: the goniometer, Leap Motion sensor, and the TactileGlove. Utilizing the hand pose information from the Leap Motion sensor and both finger and palm force measurements from the tactile glove, the computed force across the hand region is used to determine the resultant torque at the wrist joint. However, the absolute wrist torque may not inherently capture the variability in the individual physiological conditions. Consequently, we propose using the median of the torque as a normalization factor for each subject’s torque data. The wrist angle’s state directly impacts the tendon dynamics, which, in turn, plays a pivotal role in hand injury risk.

**Fig. 5:**
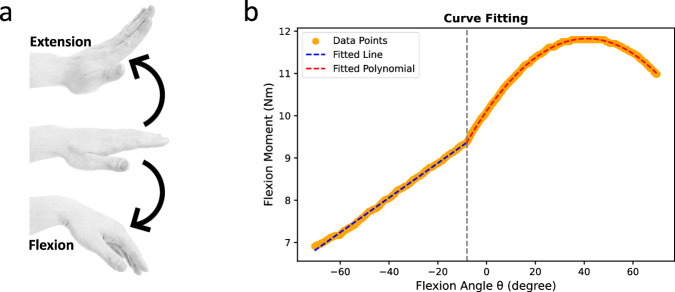
Hand pose illustration and maximum isometric wrist moments vs. flexion angle. Hand flexion/extension (**a**) and the corresponding maximum isometric wrist flexor/extensor moments (**b**) during positive flexion force is shown. The curve comprises two segments: a linear relationship in the left section and a quadratic relationship in the right section, fitted using linear and second-degree polynomial models respectively.

We obtain the relationship between the wrist flexion angle and wrist flexion moment from ref. ^35^. As shown in Fig. 5, the moment progressively increases when force is applied in the positive flexion direction as the wrist transitions from extension to flexion. It reaches its peak at approximately 40° and subsequently begins to decline. This observation implies that the maximum applicable flexion moment is constrained by the biomechanical characteristics of the hand. Consequently, it serves as an indicative parameter for assessing wrist safety during force application. Specifically, a higher achievable wrist moment at a given wrist angle corresponds to a reduced injury risk. As an extension of this principle, the inverse wrist moment at the corresponding wrist angle can be employed as a multiplicative factor for the normalized torque.

We define the BACH score as a function of the ratio of wrist torques *τ* over the median of wrist torque *τ*_median_ and a multiplicative scaling factor *α*_wr_, characterized by the ratio of the maximum wrist flexion moment, ${\max }_{\theta }{M}_{{{{{\rm{flex}}}}}}(\theta )$, to the flexion moment of a given wrist angle *θ*, *M*_flex_(*θ*):

3 $${{{{\rm{BACH}}}}}=\frac{\tau }{{\tau }_{{{{{\rm{median}}}}}}}\cdot {\alpha }_{{{{{\rm{wr}}}}}}$$

where

4 $${\alpha }_{{{{{\rm{wr}}}}}}=\frac{{\max }_{\theta }{M}_{{{{{\rm{flex}}}}}}(\theta )}{{M}_{{{{{\rm{flex}}}}}}(\theta )}$$

5 $${M}_{{{{{\rm{flex}}}}}}(\theta )=\left\{\begin{array}{ll}0.041\theta +9.696,\hfill &\,{\mbox{for}}\,\,-{90}^{\circ } \, < \, \theta \, \le -{8}^{\circ }\\ -0.001{\theta }^{2}+0.083\theta +10.110,&\,{\mbox{for}}\,\,-{8}^{\circ } \, < \, \theta \, < \, {90}^{\circ }\hfill\end{array}\right.$$

The parameters for the piecewise function *M*_flex_(*θ*) are derived using curve fitting on the graphical data presented in ref. ^35^.

### Machine learning models

Machine learning (ML) models are selected based on the similarity of the modeling technique to the risk scoring mechanism. Decision trees consist of branches that assign outputs *y* based on optimized thresholds (*β*) of the feature (*x*) values in the data:

6 $$\left\{\begin{array}{ll}y\leftarrow {y}_{1}\quad &{{{{\rm{if}}}}}\,x \, < \, \beta \\ y\leftarrow {y}_{2}\quad &{{{{\rm{if}}}}}\,x\ge \beta \end{array}\right..$$

Here, the desired output is the RULA risk score, and *x* are the upper-body posture and joint angles, therefore the decision tree mimics the incrementation of RULA scores based on predetermined threshold of joint angles. In doing so, decision trees mimic the complex RULA lookup table, but also offer more interpretability of the specific force-motion combinations directly affecting risk. We use a gradient boosted classifier, which uses ensembles of decision trees to predict risk. Specifically, gradient boosted classifiers train a sequence of decision trees, starting with a simple decision tree with high bias and low variance. Sequentially, more complex decision trees with lower bias and higher variance are added, resulting in models which are less prone to overfitting while having adequate complexity. At each *n*th stage of training, a new estimator *h*_*n*_(*x*) is added to minimize the residual error, *y* − y^*^, between the output of the current model, *y* = *F*_*n*_(*x*), and the true risk, y^*^, as follows:

7 $${F}_{n+1}(x)={F}_{n}(x)+{h}_{n}(x)={y}^{* }$$

When designing the predictors for HAL, the model needs to account for it being a time windowed score that uses a history of hand force inputs. Recurrent Neural Networks (RNN) use a hidden layer that is updated with each force input, and, therefore, has a memory that can capture the temporal dynamics of the input forces. We train Gated Recurrent Units (GRUs), a type of RNN^36^, which can handle longer input sequences than RNNs with traditional activation functions such as the hyperbolic tangent. GRUs, along with Long-Short-Term Memory (LSTM)^37^ networks use gating mechanisms to map sequential force inputs to outputs (HAL score). Update gates **z** and Reset gates **r** control the flow of information from the past to the future and are computed by applying *σ*, the logistic sigmoid activation function, elementwise to weighted input and previous hidden states. The weights are then updated to minimize the difference between the predicted and true HAL score:

8a $${{{{\bf{r}}}}}=\sigma ({{{{{\bf{W}}}}}}_{r}{{{{\bf{x}}}}}+{{{{{\bf{U}}}}}}_{r}{{{{{\bf{h}}}}}}_{t-1}),$$

8b $${{{{\bf{z}}}}}=\sigma ({{{{{\bf{W}}}}}}_{z}{{{{\bf{x}}}}}+{{{{{\bf{U}}}}}}_{z}{{{{{\bf{h}}}}}}_{t-1}),$$

where **x** is the input state, **h**_*t*−1_ is the previous hidden state, and **W** and **U** are weight matrices which are learned. Hidden states are computed via elementwise multiplication ( ⊙ ) of the update gates with previous hidden states:

9a $${{{{{\bf{h}}}}}}_{t}={{{{\bf{z}}}}}\odot {{{{{\bf{h}}}}}}_{t-1}+({{{{\bf{1}}}}}-{{{{\bf{z}}}}})\odot {\hat{{{{{\bf{h}}}}}}}_{t},$$

9b $${\hat{{{{{\bf{h}}}}}}}_{t}=\phi ({{{{\bf{Wx}}}}}+{{{{\bf{U}}}}}({{{{\bf{r}}}}}\odot {{{{{\bf{h}}}}}}_{t-1})).$$

Here, *ϕ* refers to the hyperbolic tangent function. The reset gate controls how much information to forget, and the previous hidden state is ignored if **r** is near zero. The update gate controls how much information to carry over, and serves to update the hidden unit as a ratio of the previous hidden state **h**_*t*−1_ and the new hidden state ${\hat{{{{{\bf{h}}}}}}}_{t}$. GRUs lack context vectors or output gates like LSTMs, but have been shown to have similar performance while being simpler to compute and train^36^. The reader is referred to the supplementary section for implementation details of the GRU and gradient boosting classifier models.

## Results

### Generalization of RULA and HAL scores to unseen participants

**Fig. 6:**
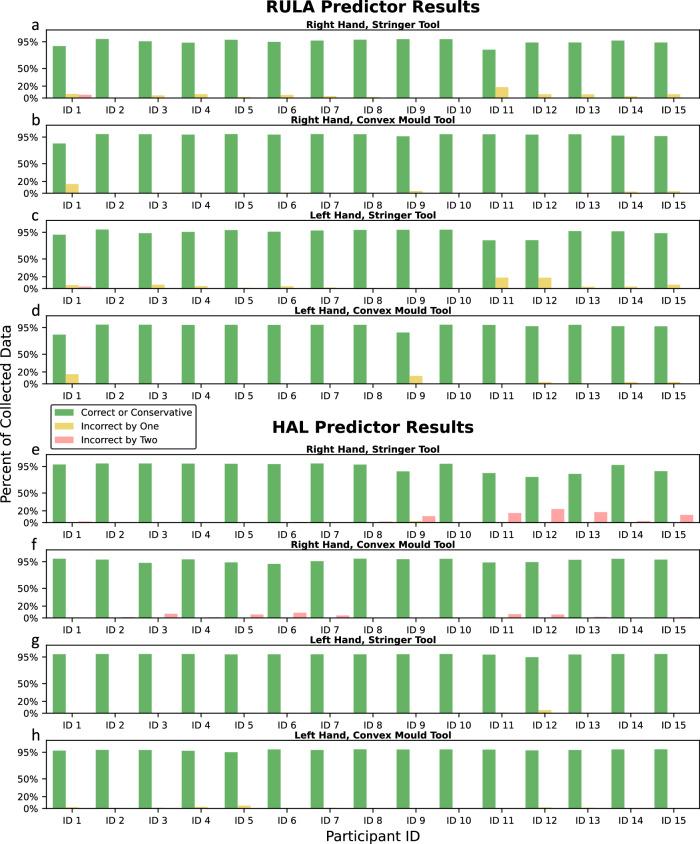
Rapid Upper Limb Assessment (RULA) and hand activity level (HAL) prediction results. Both the RULA (**a**–**d**) and HAL (**e**–**h**) predictors achieve over 95% classification accuracy for almost all the participants, across both the hands and tools. Instances of misclassification are primarily attributed to sensor malfunctions.

Automated RULA and HAL scoring from sensor data is evaluated using holdout validation, where data from one participant is reserved for testing while the model is trained on data from the remaining participants. This approach assesses the model’s ability to generalize and learn force-motion patterns associated with ergonomic risk in unseen participants. Data collection was performed using two tools with representative geometries: convex (Stringer) and concave. As all participants were right-handed, results are split by tool geometry and the hand in use to highlight any differences in scoring performance across these conditions, and shown in Fig. 6.

We categorize risk levels into three classes: low, medium, and high. This classification facilitates comparisons between HAL (0–10 scale) and RULA (1–7 scale) scores and provides practical recommendations from numerical values. To ensure conservative risk estimation, correct classifications are combined with predictions that are one class higher than the true risk level (e.g., low classified as medium, or medium classified as high). This approach promotes recommendations for ergonomic changes even in marginal cases. Figure 6 shows the percentage of data for each test participant which is correctly or conservatively classified (green), incorrectly classified by one class (low), and incorrectly classified by two classes (red) for RULA and HAL. Supplementary Tables 1 and 2 provide the actual and predicted RULA and HAL risk levels as percentages of the collected data for all participants.

The RULA predictor (Fig. 6a–d) achieves over 95% classification accuracy for nearly all participants, across both hands and tools, demonstrating its ability to model new technicians’ behavior by learning correlations between sensor data and RULA scores. The figure also reveals atypical behavior on Participant 1, Participant 11 (left and right) and Participant 12 (left) with the Stringer tool. This anomaly is primarily caused by partial occlusion of the lower section of the upper body in the camera setup, leading to inaccuracies in estimating hip position using AlphaPose and, consequently, imprecise upper body leaning angle measurements. Additionally, feature importance analysis (Supplementary Table 3) reveals that the upper extremities of the upper body are the most critical predictors of risk.

The HAL predictor (Fig. 6e–h) achieves over 95% classification accuracy for nearly all participants across both hands and tools. Notably, accuracy is higher for the left hand than the right, with atypical values observed in Participants 11–13 and, to a lesser extent, Participants 9 and 15 on the Stringer tool. Investigation revealed that force sensors on the TactileGlove saturated near the tip of the dominant (right) hand due to high local pressure, leading to incorrect force data. The GRU model, trained on these correlations, mapped the saturated data to excessively high HAL scores. When force sensor data does not saturate, the HAL predictor generalizes well, maintaining excellent accuracy across most holdout participants. In contrast, the RULA predictor shows fewer two-class misclassifications, likely due to its simpler scoring model based on angle and load thresholds, as opposed to the nonlinear functions and windowed frequency measurements used in HAL. Classification performance drops are primarily due to occlusion or sensor force saturation. Additional trials could improve holdout performance by increasing population variance and optimizing sensor configurations.

### Hand-focused ergonomic risk analysis

**Fig. 7:**
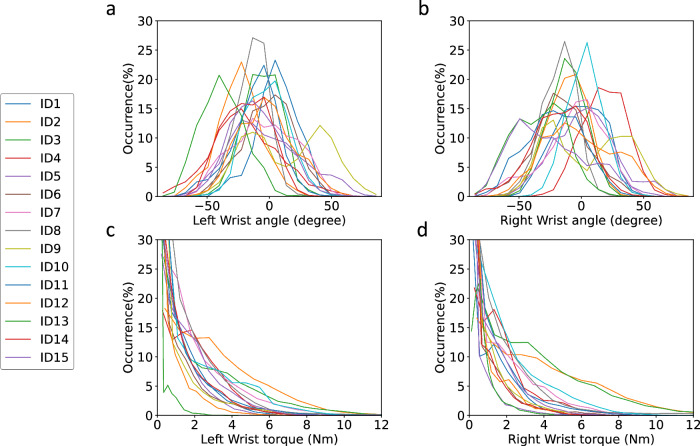
Wrist angle and torque distributions. The variations in the wrist angles (**a**, **b**) over all participants suggest different roles for the left and right hands during hand layup. The wrist torque (**c**, **d**) patterns emphasize right-handed dominance for the participants.

To assess the applicability of our BACH score, we first plot the variations in the wrist angles and torques for all the seven participants, separately for their right and left hands, in Fig. 7. We observe consistent trends in the right and left wrist torques among the participants, which highlight their individual strength disparities. To determine which hand exhibits higher torque values, we applied the Mann–Whitney U test^38^. The null hypotheses was *H*_0_: “The two torque distributions are identical”, and the alternate hypothesis was *H*_1_: “The right-hand torque distribution is greater than the left-hand distribution”. The test was conducted on *N* = 1.4 × 10^6^ samples, yielding *U* = 1.07 × 10^12^, *p* = 7.7 × 10^−132^, confirming a significantly higher occurrence of elevated torque values in the right wrists as compared to the left wrists. This result aligns with the right-handed dominance observed in all the study participants. Additionally, the wrist angles show differences in how the left and right hands are positioned, indicating that the two hands play different roles during hand layup tasks. We then select four representative sections from the hand layup trials to illustrate the characteristic patterns in the RULA, HAL, and BACH assessments. These sections portray the temporal alignment of the three distinct evaluation metrics, providing a more comprehensive visual assessment of the subjects’ hand layup movements.

**Fig. 8:**
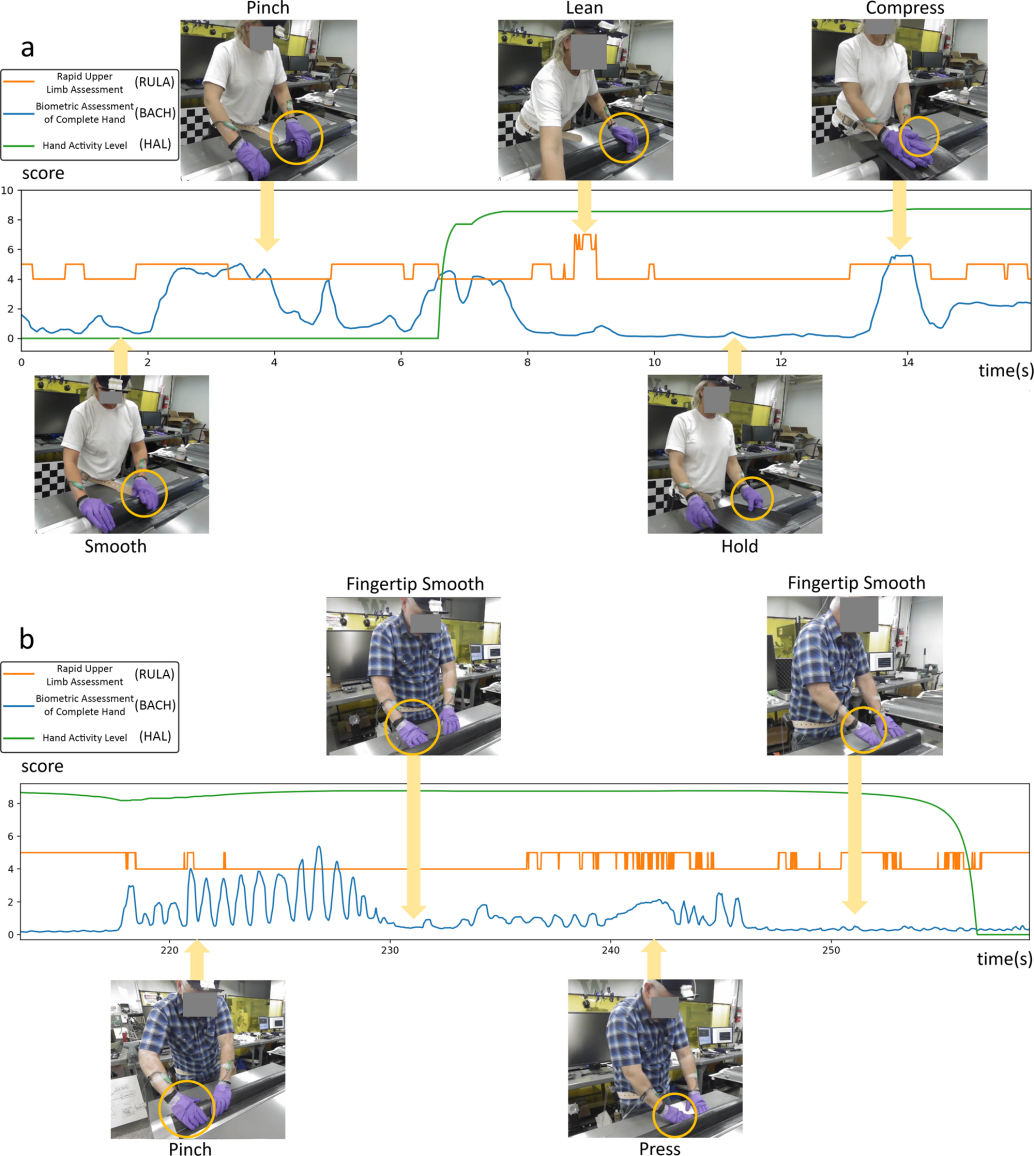
Comparative analysis of hand motion-based ergonomic scores. RULA, HAL, and BACH scores are shown from two selected trials along with the corresponding frames of interest from the digital camera. **a** BACH progresses from low scores during initial layup to peaks during pinching. RULA rises with poor posture mid-task, while BACH drops during material placement before rising again with final conforming force, and HAL shows delayed risk responses to hand activity. **b** BACH score fluctuates: high during pinching, low during smoothing, rising with pressing, then decreasing with return to smoothing, RULA maintains medium risk with minor fluctuations and HAL score exhibits an offset delayed response.

In Fig. 8, the top section illustrates a progression from gentle material rubbing with low metric scores to material pinching, resulting in higher wrist torques. As the operator leans forward using wrist support, RULA reaches high risk while BACH drops due to force alignment through the wrist. The final stages show sustained HAL elevation despite decreasing finger force, concluding with two-handed pressing that peaks BACH scores. Throughout, RULA fluctuates with posture changes while HAL maintains stable trends due to its computational parameters. BACH provides more detailed real-time assessment, complementing HAL’s limited temporal resolution. In the bottom section, the operator begins with repetitive pinching motions to conform material to the curved tool surface, causing oscillations in the BACH score. The process then shifts to fingertip smoothing at the tool’s edge, reducing force and lowering BACH scores. Increased finger pressing afterward raises the BACH score to a moderate level. Next, edge manipulation with the fingertips reduces the palm-force angle and shortens the wrist torque lever arm, lowering BACH scores. HAL then shows a delayed response to the decrease in hand activity intensity, attributed to its thresholding and windowing computation method. Throughout these activities, RULA remains at medium risk levels due to stable upper-body posture.

**Fig. 9:**
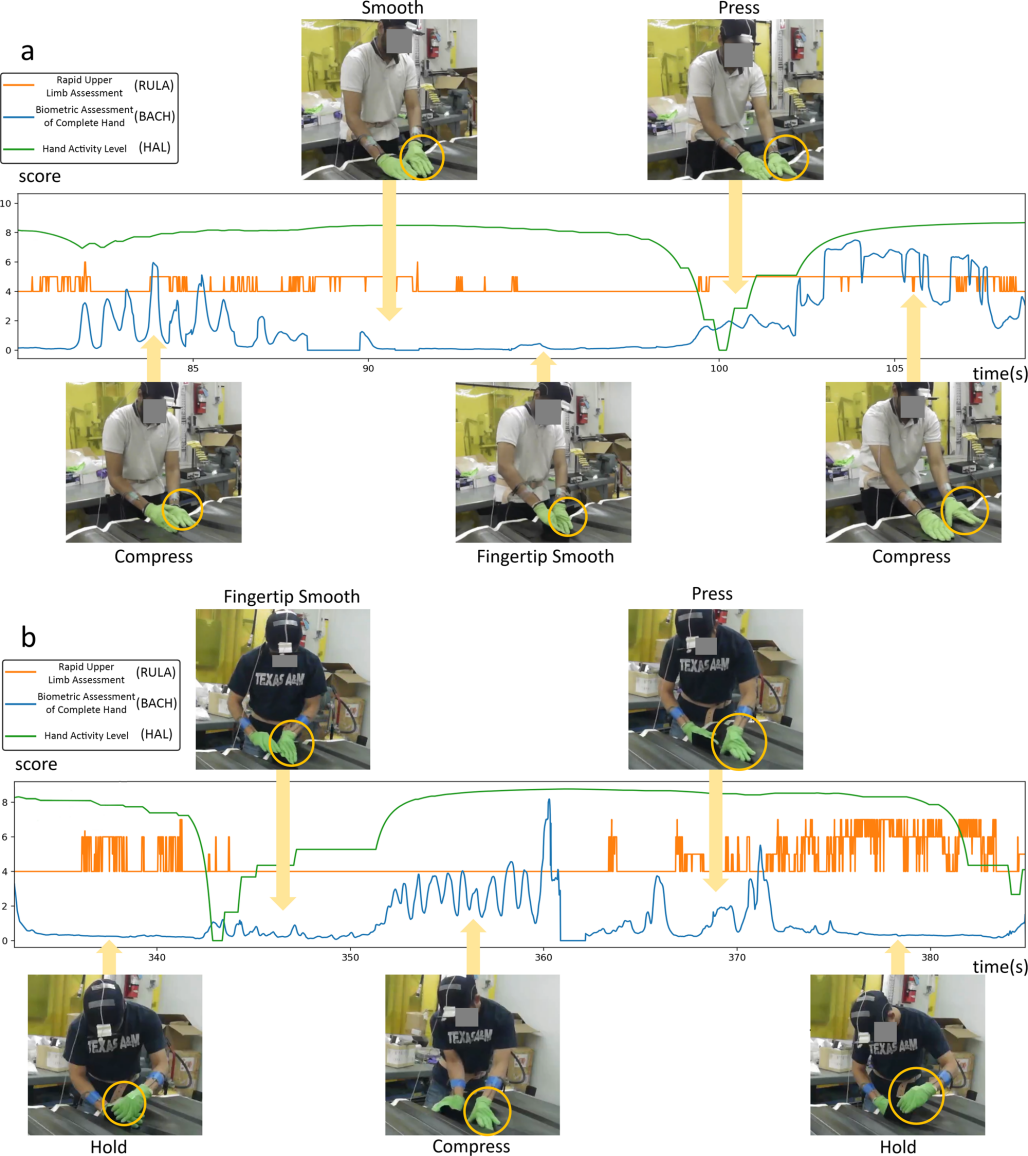
Additional comparative analysis of hand motion-based ergonomic scores. RULA, HAL, and BACH scores are shown from two selected trials along with the corresponding frames of interest from the digital camera. **a** BACH score fluctuates through task sequence: high during pressing, low during smoothing, rising with fingertip work, increasing with whole-hand pressure, and peaking with forward-leaning body weight application. **b** Task progresses from minimal wrist torque during material holding, to fingertip precision work, then whole-hand pressure, before shifting to lighter thumb pressing and final pinching motion. HAL shows delayed response to movement intensity, while RULA reflects upper body engagement.

The top section of Fig. 9 demonstrates a dynamic sequence of hand activities: BACH score initially fluctuates with repetitive pressing, then significantly drops during hand surface smoothing in the second frame. The score rises again as the operator employs fingertip precision for narrow groove fitting, followed by increased whole-hand pressure for material conformity. BACH peaks in the final frame when the operator leans forward, channeling body weight through hand pressure. Throughout these transitions, HAL demonstrates a consistent pattern of delayed response to decreasing movement intensity, while RULA variations specifically track the operator’s forward-leaning postures and their associated risks. The bottom section exhibits a distinct progression of hand movements: starting with minimal wrist torque during left-hand material holding, then transitioning to precise fingertip manipulation in narrow grooves. This is followed by whole-hand pressure application with added body weight, before shifting to lower-intensity thumb-dominant pressing. The sequence concludes with a return to material holding motion to support right-hand layup activities. As in the top section, HAL shows delayed responses to two distinct decreases in movement intensity, while RULA scores correspond directly to changes in upper body weight engagement throughout the layup process.

Visualizations of the two layup sections highlight the utility of the RULA score for assessing overall body posture risk, while the HAL and BACH metrics provide a more detailed focus on hand region analysis. Although the HAL score captures hand activity over extended timeframes, it lacks the precision for immediate, detailed hand risk assessment. The BACH score addresses this limitation, offering a more robust and nuanced evaluation of hand risk. Consequently, it adds an alternative dimension that could significantly enhance hand risk monitoring.

## Discussion

Our sensor-driven ergonomic scoring framework is of immediate applicability in numerous common manufacturing tasks involving human grasp, pressure and smoothing. The developed BACH score in particular, quantifies hand and digit activity in granular detail not afforded by other metrics (HAL and RULA). Given the prevalence of dexterous activity in manufacturing and the need for safe, efficient automation of repetitive tasks, this framework opens several promising directions for future work. Data-driven and learning-based approaches can further characterize the biomechanics of dexterous hand movements, particularly by linking hand pose and activity with indicators of tendon injury. In addition to data-driven risk scoring, such ML approaches can learn improved models of existing risk metrics such as HAL, including learning nonlinear models directly from data^39,40^, optimizing model or measurement parameters to extract maximal information^41^ or to generalize to specialized hand activities. Additionally, feature engineering of pose-force combinations can inform the development of assistive robotics, exoskeletons, and partial automation for dexterous processes. Automated decision-making in such systems could be enhanced by incorporating information on local geometry, material properties, and user-preferred movement patterns.

Notably, shop aides were not used consistently across the trials, particularly by less experienced participants. As a result, they did not significantly reduce the participants’ RULA, HAL, or BACH scores. However, the use of shop aides did impact task completion times. For the tasks where the operators utilized the shop aides over 50% of the duration, completion times were compared against those completed without any aide. A Mann–Whitney *U*-test was applied with the null hypotheses *H*_0_: “The two completion times are identical”, and the alternate hypothesis *H*_1_: “Completion times without the shop aide are greater than those with the shop aide”. Conducted on *N* = 15 samples, the test yielded *U* = 179.5, *p* = 0.003, indicating that shop aides significantly reduced the task completion times while maintaining comparable layup quality, thereby, mitigating prolonged exposure to high ergonomic risks. Nevertheless, we plan to redesign the shop aides using the BACH score and the wrist torque metric to encourage the operators to use the thumb to apply the same force instead of other fingers. This is expected to result in a lesser overall wrist torque for the same applied force, as the thumb is closer to the wrist than the other fingers. The thumb, being the topmost finger when applying force downwards, helps maintain the forearm in a neutral position, which has been shown to minimize ergonomic risks as compared to other non-neutral positions^42^.

Building on these observations, we can further refine ergonomic interventions by optimizing operator techniques and workplace setups. Targeted ergonomic adjustments can be made to both operator techniques and workplace setup in tasks like composite hand layup to lower injury risk. First, modifications to operator movements and force application are recommended, based on trials demonstrating that low-risk movements and reduced force achieve the same layup quality with less injury potential. Secondly, adjustments to the workplace, informed by analysis of upper body postures, can facilitate safe access to tool extremities without significantly raising RULA scores. By examining tool mounting angles and access points, this ergonomic assessment can also help improve the operator’s baseline posture during composite hand layup tasks.

Data collection and model training for this study were conducted using two laptop computers, integrating both sensors and computational capabilities to create a fully portable ergonomic risk assessment testbed. Our goal is to use this tool to assess ergonomic risks associated with hand-intensive activities across both manufacturing and non-manufacturing settings. The real-time inference and risk scores generated by these ML models provide technicians with immediate feedback on the most ergonomically hazardous aspects of their tasks. This feedback enables targeted adjustments—either through workplace modifications, enhanced access to the tool, expert guidance on safer techniques, or the design of shop aides that reduce exposure time and improve reach to challenging areas.

While this portable tool shows promise for real-time ergonomic risk assessment in diverse work environments, challenges remain in achieving fully reliable data. Issues like sensor saturation, missing measurements, and connectivity disruptions underscore the need for robust data handling methods to ensure model accuracy and comprehensive ergonomic insights. These failure modes pose challenges in training the most generalizable ML models, as well as in computing BACH scores that capture all the hand activity risks. In the future, it would be useful to investigate suitable data imputation techniques^43^ to fill in the missing measurements. Correlation analysis and decomposition methods such as Robust PCA^44^ and Dynamic Mode Decomposition^45^ can help impute missing data using correlated measurements from other sensors. These techniques also provide dimensionality-reduced representations of complex force-motion combinations, which can be useful for process control and automation.

Addressing these data reliability issues will support the broader goal of developing a comprehensive and objective ergonomic risk assessment. This approach represents an important step toward establishing robust injury metrics that help understand how and why operator injuries occur in manufacturing settings. By shifting from subjective, expert-based assessments to sensor-driven, objective evaluations, we can minimize errors associated with inter-rater reliability and improve accessibility. Accurately predicting injury risk remains challenging due to factors such as cumulative physical and mental fatigue. A truly comprehensive assessment will require integrating a broader range of measurable variables during evaluation.

## Supplementary information

Supplementary Information

## Data Availability

The processed hand layup experiment data are available for reference at the following URL: https://data.mendeley.com/preview/cczkvvvygw?a=80891768-f364-4e90-ab34-bfd68487de01.

## Code availability

The code repository for this project can be accessed by invitation, contact A.K. (anandkrishnan6561@gmail.com) or K.M. (kmanohar@uw.edu) for access.
