# Supplementary material for: Data-driven ergonomic risk assessment of complex hand-intensive manufacturing processes
Source: Commun Eng. 2025 Mar 12;4:45. doi: 10.1038/s44172-025-00382-w (PMC11903948; doi:10.1038/s44172-025-00382-w)
Supplement: Supplementary file 1 — Supplementary Information [file 44172_2025_382_MOESM1_ESM.pdf]

Data-Driven Ergonomic Risk Assessment of Complex Hand-Intensive Manufacturing Processes: Supplementary Materials

Anand Krishnan[1,†], Xingjian Yang[1,†], Utsav Seth[1], Jonathan M. Jeyachandran[2], Jonathan Y. Ahn[2], Richard Gardner[2], Samuel F. Pedigo[2], Adriana (Agnes) W. Blom-Schieber[1,2], Ashis G. Banerjee[1,3,*], and Krithika Manohar[1,*]

[1]Department of Mechanical Engineering, University of Washington, Seattle, WA, USA
[2]The Boeing Company, Everett, WA, USA
[3]Department of Industrial & Systems Engineering, University of Washington, Seattle, WA, USA
[†]These authors contributed equally
[*]Corresponding authors: Ashis G. Banerjee, ashisb@uw.edu; Krithika Manohar, kmanohar@uw.edu

Supplementary

Details of ML models

Gated Recurrent Units (GRUs) [1], a type of Recurrent Neural Network (RNN), are used to predict our time-dependent HAL score, which is implemented in Pytorch. The reset gate r_t, update gate z_t, new gate n_t, and the candidate hidden state h_t are computed componentwise as follows (equivalent to Equations (8-9) in the main text

$$\begin{aligned} r_t &= \sigma(x_t W_{ir} + h_{t-1} W_{hr} + b_r) \\ z_t &= \sigma(x_t W_{iz} + h_{t-1} W_{hz} + b_z) \\ n_t &= \tanh(W_{in} x_t + b_{in} + r_t \odot (W_{hn} h_{t-1} + b_{hn})) \\ h_t &= (1 - z_t) \odot n_t + z_t \odot h_{t-1} \end{aligned}$$

where x_t is the input at time t, $h_{t-1}$is the hidden state at time $t-1$ or the initial hidden state at $t = 0$. σ is the sigmoid function, and $\odot$ denotes the Hadamard product. The W and b terms denote the weight matrices and biases respectively, which are updated iteratively to improve prediction performance. We use a multilayer GRU, therefore the input $x_t^{(l)}$ of the l-th layer ($l \geq 2$) is the hidden state $h_{t-1}^{(l-1)}$ of the previous layer.

The model was also developed in Python 3.11, using PyTorch and PyTorch Lightning developed for GPU hardware accelerators and an Nvidia-CUDA compatible laptop with an RTX 4080 GPU. Here are the salient features of the model, identified after searching over possible structures and hyperparameters via grid search.

- **Structure:** Sequentially, the first layer is a Gated Recurrent Unit (*torch.nn.GRU*) with ($input\_size, hidden\_size, num\_layers$) = (6,10,3), with 6 inputs referring to the instantaneous force on the five fingers and the palm section. This is followed by a dense layer (*torch.nn.Linear*) with ($in\_features, out\_features$) = (10,90), followed by another dense layer (*torch.nn.Linear*) with ($in\_features, out\_features$) = (90,1) which produces HAL as output.

- **Optimizer and Loss Function:** The ADAM optimizer[2] (*torch.optim.Adam*) is used with a learning rate, $lr = 1e - 3$. Huber Loss is used as our Loss Function (*torch.nn.HuberLoss*) with $(delta, reduction)$ = (1,'mean').

- **Training and Early Stopping:** This model is trained for a maximum of 25 epochs with a batch size of 1024. The increased batch size resulted in a significant increase in accuracy, suggesting that the increased variance of bigger batches can more accurately capture force trends corresponding to HAL scores. We have also implemented Early Stopping $(pytorch\_lightning\_callbacks.EarlyStopping)$ with $(monitor, patience) = (train\_loss, 3)$. This moves the model from inference to training when the training loss hasn't improved in the last 3 epochs, resulting in less overfitting to the training data.

The Gradient Boosting Classifier is a state-of-the-art classifying technique that uses multiple weak learners to produce a prediction. This technique produced the best results when trained using the angles formed by the upper body as input and the corresponding RULA scores as output. This model was developed in Python 3.11 and uses the Sklearn and XGBoost packages. XGBoost is used for GPU-based hardware acceleration during training, and its Sklearn API is used to interface with Sklearn's functions for hyperparameter optimization and model selection. The hyperparameters were tuned initially over the entire parameter space with a randomized search, and fine-tuned with a grid search. All except one participant's data is used for training, with the results being demonstrated on the held out participant, similar to the GRU model. We note that the cameras are able to see more of the technician in the Convex Mold Tool compared to the Stringer Tool, due to the smaller size of the tool. Here are the details of this model.

- **Structure:** We used a pipeline (*sklearn.pipeline.Pipeline*) of a standard scaler (*sklearn. preprocessing.StandardScaler*), and a gradient boosting classifier (*xgboost.XGBClassifier* with (tree_method,sampling_method) = ('gpu_hist','gradient_based').

- **Hyperparameters and Loss:** We set (n_estimators,max_depth,learning_rate,gamma, min_child_weight,max_leaves) = (29,6,0.018,0.12,1,27) after performing an extensive coarse and fine grid search. We use the F1 score (*sklearn.metrics.f1_score*) as the loss function.

Pose Estimation Accuracy Verification

We conducted an experimental investigation aimed at assessing the accuracy of upper body pose estimation using multiple inertial measurement units (IMUs). These IMUs, each comprising an accelerometer and a magnetometer, were strategically positioned on the participant's body at three key locations: the back, upper arm, and lower arm. The accelerometer's role in this configuration was to accurately measure the roll and pitch angles, while the magnetometer was utilized to ascertain the yaw angle. By integrating the data from these two sensors, we were able to precisely calculate the angular position of different body segments. Specifically, our data collection focused on measuring the inclination of the back, the angular relationship between the upper arm and the torso, and the angle formed at the elbow between the lower and upper arms. This approach allowed for a detailed analysis of upper body movements and the evaluation of the accuracy of pose estimation in various postures. A visual representation of this setup is provided in Fig. 1.

In the experimental phase, participants were initially positioned in a neutral stance, characterized by an upright posture and arms resting beside the body. This baseline position was critical for calibrating subsequent measurements, a process essential for achieving precise taring. Subsequently,

participants transitioned into various body poses, each maintained for a prolonged duration to facilitate the attainment of consistent and stable readings. The experiment encompassed a variety of poses to evaluate the efficacy of the body pose estimation methodology.

After completing the experiment, direct readings were acquired from IMUs, alongside the 3D upper body pose data reconstructed from AlphaPose. The data from both the IMUs and AlphaPose were synchronized to maintain temporal consistency. Following this, periods during which the subjects held steady poses were identified and extracted for detailed analysis. During these intervals, the average angle of each body section was computed. This was followed by a comparative analysis between the IMU-derived body pose and the reconstructed pose data from AlphaPose. The findings indicated that this method was generally effective in estimating body poses. However, it is important to highlight that the precise placement of on-body sensors presented a significant challenge. This difficulty was primarily attributed to the potential alteration of sensor positions caused by muscle movements during different poses, a factor that could adversely impact the accuracy of the collected data.

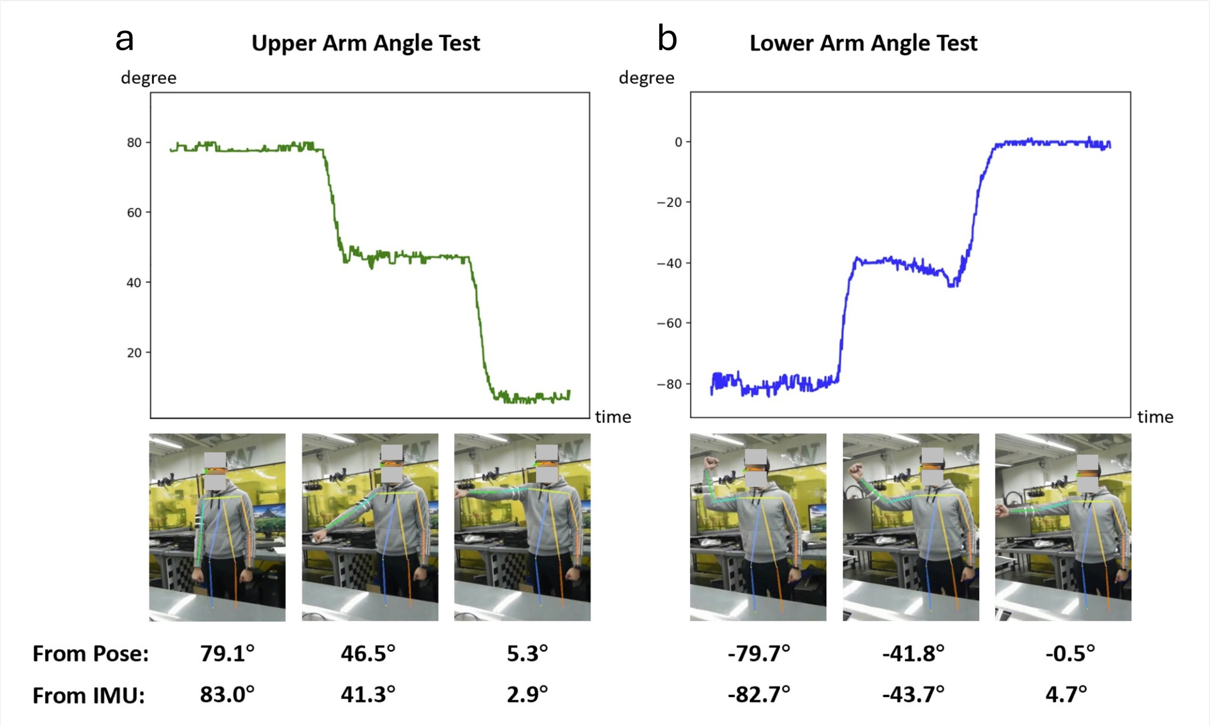

Fig. 1 | Accuracy Comparison Between Pose Estimation and IMU Measurement for Upper and Lower Arm Angle. The curve is the angle obtained from the 3D estimated pose: (a) angle between upper arm and body, (b) angle between lower arm and upper arm. The number at the bottom shows the average arm angle from both 3D pose and IMU reading during each time period, it is evident that the pose estimation process yields generally reliable results with respect to accuracy.

Additional Results

This section has the detailed risk level results for the HAL and RULA predictors respectively. Table 1 shows the RULA model holdout validation accuracy in percentage. The risk levels are segmented

as low, medium and high based on the risk levels, namely, low (0-3), medium (4,5), and high (6,7). Table 2 shows the HAL model holdout validation accuracy in percentage. We use three classes for the risk levels, namely, low ($0 < HAL < 4$), medium ($4 < HAL < 7$), and high ($HAL > 7$). The results have been split based on the left and right hand, and also based on the tools. The results mainly show the classifier generalizes well to new participants.

Table 3 shows the ranking of the most important features for a RULA classifier trained on the 3D body coordinates. This table thus shows the most important features the classifier uses to predict the RULA score, ranked using the Maximum Relevance Minimum Redundancy algorithm[3]. The L, R, or Mid designations refer to the left or right side of the body, or their average respectively. Channels 1 and 2 of the goniometer represent flexion and extension angles of the wrist. This table highlights the importance of the upper extremities in determining risk, namely, the shoulder and elbow positions, along with the wrist flexion/extension angle.

**Right,Stringer Tool Participant ID**	Correctly Classified	True=Low Predicted=Medium	True=Low Predicted=High	True=Medium Predicted=High	True=Medium Predicted=Low	True=High Predicted=Medium	True = High Predicted=Low
1	81.31	1.45	0.82	4.86	6.08	0.79	4.7
2	99.52	0.1	0	0.03	0.12	0.23	0
3	93.7	0	0	2.09	0	4.21	0
4	91.94	0.96	0	0.51	0.33	6.22	0.05
5	96.69	0.86	0	0.73	0.12	1.61	0.01
6	91.6	0.58	0.12	2.51	0.93	4.24	0.02
7	96.07	0.14	0	0.75	0.05	2.96	0.03
8	90.94	0	0	7.53	0.08	1.43	0.01
9	95.07	0.03	0	4.15	0.01	0.74	0
10	98.92	0.05	0	0.37	0	0.66	0
11	78.03	0	0	3.5	0	18.47	0
12	88.51	0	0	5.22	0	6.27	0
13	93.42	0	0	0.41	0	6.17	0
14	95.91	0.15	0	0.92	0.13	2.88	0.01
15	93.56	0	0	0.19	0	6.25	0

**Right,Convex Mold Tool Participant ID**	Correctly Classified	True=Low Predicted=Medium	True=Low Predicted=High	True=Medium Predicted=High	True=Medium Predicted=Low	True=High Predicted=Medium	True = High Predicted=Low
1	82.55	1.34	0.02	0.12	15.69	0.05	0.23
2	99.96	0	0	0	0	0.04	0
3	99.67	0.12	0	0	0.01	0.2	0
4	97.97	1.06	0	0.01	0.53	0.38	0.04
5	99.82	0.09	0	0	0.01	0.08	0
6	98.41	0.65	0	0.05	0.5	0.4	0
7	99.89	0.09	0	0	0.01	0.01	0
8	98.16	1.55	0	0	0.26	0.02	0.02
9	94.76	0	0	1.49	0	3.75	0
10	99.72	0	0	0.08	0.03	0.17	0
11	97.8	1.55	0	0.28	0.18	0.18	0
12	98.91	0.18	0	0	0.17	0.73	0.01
13	90.17	0	0	9.42	0	0.42	0
14	95.26	1.76	0	0.35	0.3	2.32	0
15	96.03	0	0	0.58	0	3.38	0

**Left,Stringer Tool Participant ID**	Correctly Classified	True=Low Predicted=Medium	True=Low Predicted=High	True=Medium Predicted=High	True=Medium Predicted=Low	True=High Predicted=Medium	True = High Predicted=Low
1	84.8	1.64	2.17	4.49	5.07	0.77	1.07
2	99.63	0.14	0	0.02	0.05	0.15	0
3	91.22	0	0	2.28	0	6.5	0
4	94.28	0.94	0	0.52	0.34	3.91	0.02
5	96.03	1.33	0	1.42	0.07	1.14	0
6	92.59	1.33	0.01	2.17	0.5	3.3	0.11
7	97.43	0.14	0	0.54	0.01	1.84	0.04
8	87.07	0	0	11.99	0.04	0.85	0.05
9	95.1	0.04	0	4.03	0	0.82	0
10	98.87	0.04	0	0.47	0	0.62	0
11	75.68	0	0	5.87	0	18.45	0
12	79.21	0	0	2.37	0	18.43	0
13	81.29	0	0	15.69	0	3.02	0
14	95.9	0.11	0	0.7	0.1	3.16	0.03
15	93.45	0.01	0	0.06	0	6.48	0

**Left,Convex Mold Tool Participant ID**	Correctly Classified	True=Low Predicted=Medium	True=Low Predicted=High	True=Medium Predicted=High	True=Medium Predicted=Low	True=High Predicted=Medium	True = High Predicted=Low
1	81.44	1.45	0.34	0.21	16.24	0.01	0.3
2	100	0	0	0	0	0	0
3	99.75	0.1	0	0.01	0	0.13	0
4	97.85	1.56	0	0.11	0.34	0.09	0.05
5	99.64	0.1	0	0	0	0.26	0
6	98.22	1.19	0	0.06	0.2	0.33	0
7	99.48	0.11	0	0.13	0	0.27	0
8	97.94	1.71	0	0	0.17	0.09	0.09
9	74.42	0	0	12.27	0	13.31	0
10	99.68	0	0	0.16	0.03	0.13	0
11	97.33	1.84	0	0.35	0.11	0.37	0
12	96.89	0.23	0	0.19	0.26	2.41	0.01
13	92.94	0	0	6.74	0	0.32	0
14	95.18	1.84	0	0.23	0.26	2.49	0
15	96.09	0	0	1.06	0	2.84	0

Table 1: **RULA Model Holdout Validation Accuracy.** Table showing prediction accuracy of the best performing RULA model (in percent) using XGBoost architecture using holdout validation. The results are the predictions of the model when given the previously unseen sensor data of the current participant while the remaining participants' data and RULA scores are used to train the model. The results are displayed for the right and left hands for both tools used in data collection.

**Right,Stringer Tool Participant ID**	Correctly Classified	True=Low Predicted=Medium	True=Low Predicted=High	True=Medium Predicted=High	True=Medium Predicted=Low	True=High Predicted=Medium	True = High Predicted=Low
1	97.79	0	1.79	0.41	0	0	0
2	99.98	0	0.02	0	0	0	0
3	99.97	0	0.03	0	0	0	0
4	98.64	0.03	0.17	1.16	0	0	0
5	98.9	0.34	0.55	0.21	0	0	0
6	96.79	0.12	1.14	1.94	0	0	0
7	99.38	0	0.08	0.54	0	0	0
8	96.48	0	1.93	1.59	0	0	0
9	60.05	2.91	10.89	23.56	0	2.59	0
10	97.23	1.15	0.53	1.09	0	0	0
11	82.74	0	16.25	1.01	0	0	0
12	56.93	0.13	22.87	20.08	0	0	0
13	65.73	0	17.64	16.63	0	0	0
14	96.43	0.08	2.66	0.83	0	0	0
15	77.25	0	13.03	9.72	0	0	0
**Right,Convex Mold Tool Participant ID**	Correctly Classified	True=Low Predicted=Medium	True=Low Predicted=High	True=Medium Predicted=High	True=Medium Predicted=Low	True=High Predicted=Medium	True = High Predicted=Low
1	99.77	0	0.01	0.22	0	0	0
2	96.56	0	1.45	1.99	0	0	0
3	87.55	0.34	7.07	5.04	0	0	0
4	98.14	0	1.16	0.7	0	0	0
5	86.07	2.08	5.6	5.72	0.39	0.14	0
6	82.01	2.47	8.68	6.84	0	0	0
7	94.71	0	4.22	1.07	0	0	0
8	99.23	0	0	0.77	0	0	0
9	97.58	0.15	0.74	1.53	0	0	0
10	97.87	0.41	0	1.67	0	0.05	0
11	90.16	0.59	6.38	2.87	0	0	0
12	91.52	0	5.74	2.74	0	0	0
13	93.07	0.15	1.81	4.97	0	0	0
14	99.6	0.16	0.15	0.09	0	0	0
15	96.53	0.65	1.51	1.3	0	0	0
**Left,Stringer Tool Participant ID**	Correctly Classified	True=Low Predicted=Medium	True=Low Predicted=High	True=Medium Predicted=High	True=Medium Predicted=Low	True=High Predicted=Medium	True = High Predicted=Low
1	99.55	0.06	0.01	0.02	0.02	0.34	0
2	99.98	0	0.02	0	0	0	0
3	99.97	0	0.03	0	0	0	0
4	99.94	0	0	0	0.01	0.04	0
5	99.22	0	0	0.01	0.07	0.7	0
6	99.45	0.02	0	0	0.48	0.05	0
7	99.45	0.01	0	0	0.06	0.48	0
8	98.97	0.05	0	0.5	0.01	0.47	0
9	92.78	2.31	0.15	4.61	0	0.15	0
10	99.8	0.11	0	0.06	0	0.03	0
11	97.86	1.07	0	0.01	0.49	0.57	0
12	94.6	0.02	0	0.03	0.73	4.62	0
13	97.74	0.19	0.1	1.23	0.64	0.1	0
14	99.29	0.07	0	0.63	0	0.01	0
15	97.56	1.41	0	0.93	0	0.1	0
**Left,Convex Mold Tool Participant ID**	Correctly Classified	True=Low Predicted=Medium	True=Low Predicted=High	True=Medium Predicted=High	True=Medium Predicted=Low	True=High Predicted=Medium	True = High Predicted=Low
1	96.97	0.06	0.01	0.79	1.16	1.01	0
2	98.68	0.15	0.01	0.08	0.12	0.96	0
3	97.74	0.81	0.01	0.16	0.11	1.17	0
4	97.36	0	0	0	0.76	1.87	0
5	94.86	0.06	0	0.17	2.32	2.52	0.07
6	99.08	0.07	0	0.67	0.01	0.17	0
7	98.6	0.19	0	0.02	0.11	1.07	0
8	99.71	0	0	0.29	0	0	0
9	96.47	0.24	0	2.93	0.3	0.06	0
10	98.75	0.08	0	0.9	0.08	0.19	0
11	97.71	0.67	0.35	1.11	0.01	0.14	0
12	96.56	0.29	0	1.28	0.82	1.05	0
13	94.79	0.64	0	3.25	0.7	0.61	0
14	98.87	0.46	0	0.44	0.01	0.22	0
15	99.85	0.09	0	0.04	0	0.01	0

Table 2: **HAL Model Holdout Validation Accuracy.** Table showing prediction accuracy of the best performing HAL model (in percent) using GRU architecture using holdout validation. The results are the predictions of the model when given the previously unseen sensor data of the current participant while the remaining participants' data and HAL scores are used to train the model. The results are displayed for the right and left hands for both tools used in data collection.

Left RULA	Right RULA
Mid_Shoulder - Y	L_Shoulder - Y
L_Gonio - 1	R_Elbow - Z
L_Elbow - X	Mid_Shoulder - Y
L_Eye - Z	R_Elbow - X
R_Shoulder - Y	R_Gonio - 1
L_Shoulder - Y	L_eye - Z

Table 3: **RULA Classifier Feature Importance Ranking** Table showing the ranking of features by importance to the RULA scores, ranked using the MRMR algorithm[3]. This classifier used the 3D coordinates of body pose, and the goniometer data. It highlights the importance of shoulder position, wrist flexion/extension angle (given by L_Gonio - 1 and R_Gonio - 1) and elbow angle.

References

[1] Cho, K., Van Merriënboer, B., Gulcehre, C., Bahdanau, D., Bougares, F., Schwenk, H., and Bengio, Y. Learning phrase representations using rnn encoder-decoder for statistical machine translation. *arXiv preprint arXiv:1406.1078* (2014).

[2] Kingma, D. P. and Ba, J. Adam: A method for stochastic optimization. *arXiv preprint arXiv:1412.6980* (2014).

[3] Peng, H., Long, F., and Ding, C. Feature selection based on mutual information criteria of max-dependency, max-relevance, and min-redundancy. *IEEE Transactions on Pattern Analysis and Machine Intelligence* **27**(8), 1226–1238 (2005).
